# The prevalence of dyspepsia symptoms and its correlation with the quality of life among Qashqai Turkish migrating nomads in Fars Province, Southern Iran

**DOI:** 10.12669/pjms.312.6956

**Published:** 2015

**Authors:** Seyed Jalil Masoumi, Davood Mehrabani, Fariba Moradi, Najaf Zare, Mehdi Saberi-Firouzi, Zohreh Mazloom

**Affiliations:** 1 Seyed Jalil Masoumi, Nutrition and Food Sciences Research Center, School of Nutrition and Food Sciences, Gastroenterohepatology Research Center, Shiraz University of Medical Sciences, Shiraz, Iran; 2 Davood Mehrabani, Department of Pathology, Stem Cell and Transgenic Technology Research Center, Shiraz University of Medical Sciences, Shiraz, Iran; 3 Fariba Moradi, Shiraz Geriatric Research Center, Office of Vice Chancellor for Health, Shiraz University of Medical Sciences, Shiraz, Iran; 4 Najaf Zare, Department of Biostatistics, Shiraz University of Medical Sciences, Shiraz, Iran; 5 Mehdi Saberi-Firouzi, Digestive Diseases Research Institute, Shariati Hospital, Tehran University of Medical Sciences, Tehran, Iran; 6 Zohreh Mazloom, School of Nutrition and Food Sciences, Shiraz University of Medical Sciences, Shiraz, Iran

**Keywords:** Prevalence, Dyspepsia, Quality of life, Nomads, Iran

## Abstract

**Objectives:**

To determine the prevalence of dyspepsia and its correlation with quality of life in Fars Qashqai Turkish migrating nomads from Southern Iran.

**Methods:**

During 2010 we enrolled 397 Qashqai migrating nomads from Southern Iran who were 25 years of age or older. Participants completed a questionnaire that consisted of demographic factors, lifestyle data, gastrointestinal symptoms, and the Short-Form 36 Health Survey (SF-36) questionnaire.

**Results:**

There was a 48% prevalence of dyspepsia symptoms among participants. The prevalence was higher among females, those less than 35 years of age, married participants, and those with a low body mass index (BMI). The correlation between dyspepsia and quality of life was significant. Dyspeptic patients were classified into ulcer-like (27.9%), dysmotility-like (26.2%), and unspecified (45.9%) groups. A significant correlation existed between dyspepsia symptoms and consumption of dairy products, drinking water and tea before and after meals, smoking, dysphagia, reflux, heartburn, and use of non-steroid anti-inflammatory drugs and acetaminophen.

**Conclusion:**

The high prevalence of dyspepsia in Qashqai nomads necessitates educational health programs for the migrating tribes in order to decrease prevalence of this disease.

## INTRODUCTION

Dyspepsia is a syndrome that consists of burning, discomfort, epigastric pain, early satiety, fullness, belching, nausea and vomiting.^1^ Uninvestigated dyspepsia has been reported in 8% to 30% of Asians, whereas 8%-23% report the presence of functional dyspepsia.^2^ Although dyspepsia is a worldwide concern, the majority of reports arise from Western countries. Dyspepsia is mostly organic in nature among populations from developing countries, whilst functional dyspepsia is more common in Western nations.^3^ Cultural differences, socio-psychological issues, gastrointestinal infections such as *Helicobacter pylori*, dietary factors, the presence of organic diseases such as peptic ulcer and gastric cancer responsible for dyspeptic symptoms can affect the epidemiology of this disease.^4^

Generally accepted as a non-life-threatening disorder, most cases of dyspepsia do not necessitate surgery, nor is a reduction in survival observed.^2^ These patients experience significant levels of abdominal dyspepsia^5^ in addition to considerable anxiety and demonstrable abnormal healthcare-seeking behaviors.^6^,^7^

The prevalence of dyspepsia varies among studies and may be attributed to various definitions used in these studies.^8^ Although this entity is non-life-threatening, the symptoms are long-lasting^9^ with increased medical costs that interfere with daily activities and affect quality of life.^10^ A recent review of the general population also refers to the impact of dyspepsia on quality of life.^11^

We previously studied the prevalence of dyspepsia in Fars Province, Southern Iran in the general population (29.9%) and determined its correlation with demographic factors and lifestyle.^12^ The Qashqai migrating nomads of Fars Province were the subjects of two studies that evaluated the correlation between quality of life and gastro-esophageal reflex disease (GERD). In these studies, we reported prevalences of 28.5% for GERD (13) and 11.8% for irritable bowel syndrome (IBS).^13^,^14^ There was no study on dyspepsia symptoms among the Qashqai migrants in Southern Iran. Therefore, the present study sought to determine the prevalence of dyspepsia and its correlation with quality of life.

## METHODS

During 2010, we have randomly selected 397 Qashqai Turkish migrating nomads according to the last national census data.^15^ These tribes reside in tents with their domestic animals and usually migrate a distance of approximately 500 kilometers from their summer residences to their winter quarters. The nomads speak Turkish. The studied group is residents during the summer of Fars Province, Southern Iran.

Enrolled subjects were 25 years of age or older and of both genders. A team of previously trained interviewers completed the questionnaire that consisted of three parts: (i): socio-demographic details that included variables such as age, gender, and marital status; (ii): a validated Persian translation of the Short-Form 36 Health Survey (SF-36) questionnaire for the Iranian population^16^ and (iii): the Rome II questionnaire for dyspepsia symptoms.^12^

Sociodemographic variables included gender, age, educational level, marital status, habitat (summer or winter quarters), lifestyle that included physical activity (at least 30 min/week or sufficient to produce adequate sweating), cigarette smoking, dietary habits, coffee and tea consumption, alcohol, biological characteristics such as body mass index (BMI), and the use of non-steroid anti-inflammatory drugs (NSAIDs) and low dose aspirin. We defined BMI as: fasting weight (kg) divided by height (m^2^) which resulted in five categories: thin (<18 kg/m^2^), normal (18-24.9 kg/m^2^), overweight (25-29.9 kg/m^2^), obese (30-40 kg/m^2^) and very obese (>40 kg/m^2^). Dyspepsia was defined and scored as epigastric or upper abdominal symptoms (pain or discomfort) that lasted for at least three months continuously or intermittently during the past year.^12^ According to the predominant symptom, we categorized dyspepsia into three entities. Ulcer-like dyspepsia was defined as localized pain aggravated by hunger alleviated with food consumption or antacids, or pain that resulted in nocturnal awakening, or the absence of pain for at least two weeks followed by a recurrence of pain. Dysmotility-like dyspepsia included pain aggravated by food intake or the presence of post-prandial fullness and unspecified dyspepsia was defined as neither of the previous entities.

The Ethics Committee at Shiraz University of Medical Sciences approved the study and a written consent was provided from each participant. Statistical analysis was performed using the SPSS software (Version 11.5, Chicago, IL, USA). Based on missing observations, in statistical analysis the maximum range of data was included. Chi-square and t tests were applied for analysis and for comparison of mean, and for correlation between two variables, Pearson correlation test was used. A p-value of less than 0.05 was considered statistically significant.

## RESULTS

Three hundred ninety seven subjects were enrolled in this study. It included 39.6% male and 60.4% female participants. All participants completed the questionnaire. The prevalence of dyspepsia was 48% of study participants. There were more participants with dyspeptic symptoms in the <35 year-old age group, females, married persons, and those with low BMI values (Table-I). Dyspeptic patients were classified as ulcer-like (27.9%), dysmotility-like (26.2%), and unspecified (45.9%). As shown in Table-II, we observed significant differences in quality of life scores between subjects who suffered from dyspepsia and those with no symptoms.

**Table-I:** The prevalence rates of dyspepsia in relation to demographic data.

Variable		With dyspepsia	Without dyspepsia	p-value
		N (%)	N (%)
Age (years)	25-34	44 (27.0)	65 (38.3)	0.008
	35-44	37 (22.7)	53 (23.9)	
	45-54	32 (19.6)	45 (20.3)	
	55-64	24 (14.7)	26 (11.7)	
	≥65	26 (16.0)	13 (5.9)	
Gender	Female	112 (66.7)	143 (62.4)	0.224
	Male	56 (33.3)	86 (37.6)	
Marital status	Single	13 (7.8)	30 (13.3)	0.225
	Married	149 (89.2)	190 (84.1)	
	Other	5 (3.0)	6 (2.7)	
Body mass index (BMI)	<18.5	14 (8.5)	16 (7.1)	0.165
	18.5-24.9	81 (49.4)	136 (60.4)	
	25-29.9	47 (8.7)	53 (23.6)	
	≥30	22 (13.4)	20 (8.9)	

Other: Widowed or divorced.

**Table-II:** Effects of dyspepsia symptoms on quality of life.

Variable	With dyspepsia (N=168)	Without dyspepsia (N=229)		95% CI for mean Lower bound	95% CI for mean Upper bound	p-value
	Mean (SD)	Mean (SD)	Mean difference			
Physical functioning	73.7 (27.8)	83.9 (24.0)	10.2	75.5	81.2	<0.001
Role physical	49.2 (47.7)	78.2 (38.9)	29.0	58.8	68.4	<0.001
Bodily pain	64.5 (20.9)	77.6 (15.8)	13.1	69.2	73.4	<0.001
General health	50.5 (19.8)	64.2 (18.6)	13.7	55.3	59.7	<0.001
Social functioning	71.0 (22.1)	81.1 (19.5)	10.1	74.7	79.2	<0.001
Role Emotional	54.4 (47.6)	76.7 (39.6)	22.3	61.8	71.2	<0.001
Vitality	57.2 (18.7)	66.6 (18.7)	9.4	60.6	64.7	<0.001
Mental health	57.2 (20.3)	66.1 (19.1)	8.9	60.5	64.8	<0.001
Physical component summary (PCS)	59.5 (23.4)	76.0 (19.6)	16.5	-	-	<0.001
Mental component summary (MCS)	60.0 (22.0)	72.6 (18.7)	12.6	-	-	<0.001

Table-III shows the prevalence rates of dyspepsia symptoms in relation to lifestyle. There was a statistically significant correlation between dyspepsia and consumption of dairy products before and after meals. This statistical significance was also observed between dyspepsia and drinking water, and with consumption of tea before and after meals. There was a significant correlation observed between dyspepsia and smoking, dysphagia, reflux, heartburn, and use of NSAIDS and acetaminophen.

**Table-III:** The prevalence rate of dyspepsia in relation to lifestyle.

Variable	With dyspepsia (%)	p-value
Fried food intake		0.425
Yes	186 (56.7)	
No	15 (65.2)	
Vegetable consumption		0.122
Yes	195 (56.5)	
No	6 (85.7)	
Drinking water (before or after meals)	163 (38.7)	0.001
Before	67 (46.9)	
After	126 (63.0)	
No	8 (100)	
Dairy consumption	87 (20.9)	0.007
Before	29 (41.4)	
After	152 (59.8)	
None	18 (72.0)	
Drinking tea (before or after meal)		0.008
Before	81 (50.0)	
After	111 (62.4)	
None	9 (90.0)	
Smoking (1-6/day)		<0.001
≤3	62 (73.8)	
>3	131 (51.6)	
Drinking alcohol		0.393
Yes	4 (80.0)	
No	190 (56.2)	
Reflux symptoms		<0.001
Yes	163 (97.0)	
No	36 (20.2)	
Heart burn (moderate + severe)		<0.001
No	37 (35.2)	
Mild	75 (94.9)	
Moderate	46 (85.2)	
Severe	10 (76.9)	
Dysphagia (moderate + severe)		0.100
Yes	7 (100)	
No	159 (65.7)	
NSAIDS		0.032
Yes	114 (62.3)	
No	85 (50.9)	
Acetaminophen		0.016
Yes	95 (64.6)	
No	106 (51.7)	
Low dose aspirin		0.737
Yes	2 (66.7)	
No	199 (57.0)	

## DISCUSSION

Chronic and recurrent gastrointestinal symptoms are mostly attributed to functional gastrointestinal disorders that include functional dyspepsia.^17^ In the US, the prevalence of dyspepsia is 25%^1^ and in the UK it is 21%.^2^ Dyspepsia is prevalent among 25% of Western countries.^5^ In Korea, dyspepsia has been reported in 20.4% of the population. Having relatives with gastric cancer, education below college level and high-salt diet was associated with functional dyspepsia symptoms.^3^ In a longitudinal 10-year follow-up study, although 38.1% of subjects had complaints of dyspepsia there was no association with increased mortality.^18^ In a population-based endoscopic study in China a study reported a 2.4% prevalence of dyspepsia.^19^ However, another study from China reported an 18.4% prevalence of dyspepsia.^4^ In a rural population in Malaysia, 14.6% reported symptoms of dyspepsia.^20^

The epidemiology of dyspepsia in another Malaysian study was 24.3%.^21^ Among the Swedish general population, 16% reported the presence of functional dyspepsia.^22^ A Russian study reported 37.5% dyspepsia in the study population.^23^ In the current study, we reported a 48% prevalence of dyspepsia which closely approximated some reports, 18,23,24 but was less than other studies.^1^-^5^,^19^,^21^,^22^ The difference in prevalence between studies could be attributed to different definitions of dyspepsia and the differences in the enrolled populations.^4^,^23^

The level of education might affect the type of presentation of symptoms. Our findings agreed with studies that found a higher prevalence rate among less educated subjects.^3^,^18^ However a study by Mahadeva et al. reported the opposite findings.^20^ Although age was not shown to be predictive of dyspepsia,^23^ however we noticed a significant correlation between age and the prevalence of dyspepsia in patients younger than 35 years of age. The prevalence of malignancies and peptic ulcers was very low in this age group.

Although not life-threatening, dyspepsia symptoms interrupt daily activities and have significant association with a decline in quality of life and increased medical costs.^10^ Functional dyspepsia was associated with impaired quality of life.^11^ Psychological disturbances could influence quality of life in patients with dyspepsia. It was shown that abdominal pain and indigestion were main factors that influenced quality of life.^25^ In the current study we observed a significant association between dyspepsia and quality of life.

In Malaysian adults who underwent esophagogastroduodenoscopies for the primary indication of dyspepsia, 56.3% had functional dyspepsia with lower health-related quality of life compared to those with no functional dyspepsia.^24^ A door-to-door survey performed in a representative rural Malaysian population found that an association existed between dyspepsia in rural Malaysians and female gender, higher education level, medium-range incomes, nonsmokers, non-tea drinkers, regular analgesia use and adults with chronic illnesses. Conversely, regular tea drinking showed an inverse relationship that was associated with a lower health related quality of life.^20^ We also observed a significant relationship between drinking tea, acetaminophen use, NSAIDS, smoking and dyspepsia. Similarly, in several studies, dyspepsia was more common in females.^2^,^3^,^18^,^20^ Another study reported a nearly equal gender distribution among dyspeptic patients.^7^ However in one study, males were more affected by dyspepsia.^8^

The epidemiology of dyspepsia in a multi-ethnic Asian population in Malaysia and its impact on health-related quality of life showed that ethnicity was a risk factor for dyspepsia.^21^ Our findings denoted a higher prevalence of dyspepsia in Qashqai migrating nomads in Southern Iran. Therefore, in terms of ethnicity, our results were consistent with the results of other studies which found higher prevalence rates in ethnic populations.^17^,^21^

A study in Sweden explored the impact of functional dyspepsia by applying the Rome III definition on health-related quality of life. In the general population of Sweden, functional dyspepsia impacted all major physical, mental and social aspects of health-related quality of life which was similar to our study.^22^ A Russian study reported higher levels of dyspepsia in women (p=0.001)^23^ which was identical to the current study but the difference was not statistically significant. In the Russian study, quality of life significantly decreased in subjects with dyspepsia.^23^ A history of depression and self-reported stress was observed among 41.4% of patients with uninvestigated dyspepsia.^9^ It was shown that mental distress was more common in patients with dyspepsia.^3^,^10^,^18^-^20^,^22^,^25^ These findings supported the results of the current study.

We divided dyspeptic participants in our study into three subgroups with the following prevalences: 27.9% (ulcer-like), 26.2% (dysmotility-like), and 45.9% (unspecified). Our results were comparable with those reported in Shiraz, Iran and in England in dyspeptic patients. In these studies, 30% had ulcer-like dyspepsia and 32% had dysmotility-like dyspepsia.^3^,^6^ In another study in China, 50.2% of dyspeptic patients suffered from non-specific dyspepsia.^4^

Considering the relation with gastrointestinal disorders and functional dyspepsia, several researchers emphasized that abdominal pain, indigestion, reflux esophagitis, peptic ulcer disease or *Helicobacter pylori* infection could be factors that influenced health related quality of life.^19^-^22^,^24^ These reports were similar to the current study results. A Chinese study reported no association between dyspepsia and reflux esophagitis, peptic ulcer disease or *Helicobacter pylori* infection.^19^,^26^ The current study showed a significant correlation between reflux symptoms and dyspepsia.

Our findings showed that consumption of fried foods was correlated with dyspepsia.^3^,^18^,^20^ However for vegetables this was not statistically significant.^27^-^29^ Drinks that included tea, water and dairy products showed significant correlation with dyspepsia. This finding is in contrast with a report by Mahadeva et al.^20^ Our results also showed a statistical significance in regard to smoking which confirmed some study findings,^21^,^25^ but are contrary to the results reported from another study.^20^ Data on participants’ use of medications such as NSAIDs indicated a correlation with dyspepsia,^30^-^32^ however with low dose aspirin, there was no correlation with dyspepsia.^18^,^20^

## CONCLUSION

Dyspepsia was shown to have a relatively high prevalence in Fars Qashqai migrating nomads from Southern Iran. This disorder was associated with quality of life and lifestyle of the patients. There is a need for educational health program in these tribes to decrease the prevalence of dyspepsia, in particular on general and mental health as well as patients’ lifestyles.
